# Heyde’s Syndrome Complicating Management in a Patient With High Bleeding and Thrombotic Risks

**DOI:** 10.7759/cureus.8280

**Published:** 2020-05-25

**Authors:** Talha Ahmed, Reyaz Haque

**Affiliations:** 1 Internal Medicine, University of Maryland Medical Center, Baltimore, USA; 2 Internal Medicine/Cardiovascular Medicine/Interventional Cardiology, University of Maryland School of Medicine, Baltimore, USA; 3 Cardiology, University of Maryland Medical Center, Baltimore, USA

**Keywords:** aortic stenosis, heyde syndrome, gi bleeding, valve replacement, aquired von willbrand disease

## Abstract

The association of severe aortic stenosis and gastrointestinal (GI) bleeding is a well-known phenomenon. The pathogenesis involves an acquired deficiency of von Willebrand factor (vWF) due to shear stress resulting in alteration of vWF morphology. This results in in-appropriate cleavage of vWF multimers into smaller dysfunctional fragments. Patients with atrial fibrillation and high thrombotic risk require anticoagulation for stroke prophylaxis. We describe a case of severe intermittent GI bleeding in a patient with atrial fibrillation while being on warfarin and other novel anticoagulants. This case highlights the role of severe aortic stenosis and resultant acquired vWF deficiency in complicating decision making in patients with a need for anticoagulation due to high thrombotic risk.

## Introduction

In 1958, E.C. Heyde first described the association between severe aortic stenosis and gastrointestinal (GI) bleeding. At that time it was merely an association with no definitive explanation. However, with further progress on Heyde’s work, the pathogenesis of this syndrome, also known as Heyde’s syndrome, became more evident [1,2]. The diagnostic criteria include severe aortic stenosis, angiodysplasia with GI bleeding, and acquired von Willebrand factor (vWF) deficiency. vWF is integral in forming primary platelet plug as it adheres platelets to the endothelium. Its deficiency explains the easy bleeding observed in these patients. vWF has been postulated to play an important role in maintaining vascular integrity and defective vWF leads to dilated, ectatic submucosal vessels (angiodysplasias) [3].

Patients with atrial fibrillation and high thrombotic risk (CHADS2-VASc [congestive heart failure, hypertension, age ≥ 75 years, diabetes mellitus, stroke/ transient ischemic attack, vascular disease, age 65-74 years, sex category] of more than 4) require some form of stroke prophylaxis as their stroke risk is very high [4]. The presence of recurrent GI bleeding in these patients due to the concomitant presence of Heyde’s syndrome along with other factors makes management challenging due to a high bleeding risk score (HAS-BLED [hypertension, abnormal renal/liver function [one or two points], stroke, bleeding history or predisposition, labile international normalized ratio, elderly (> 65 years), drugs/alcohol concomitantly (one or two points)] score) [5]. To manage the aortic stenosis and its associated GI bleeding, these patients should be referred for valve replacement. In addition, the placement of a Watchman device (Boston Scientific Corp., Marlborough, MA) should also be considered to provide optimal anticoagulation for stroke prophylaxis to these patients. Our case entails the course of a patient with a similar clinical presentation. The hospital course is described, and the management rationale is reviewed. 

## Case presentation

**Figure 1:**
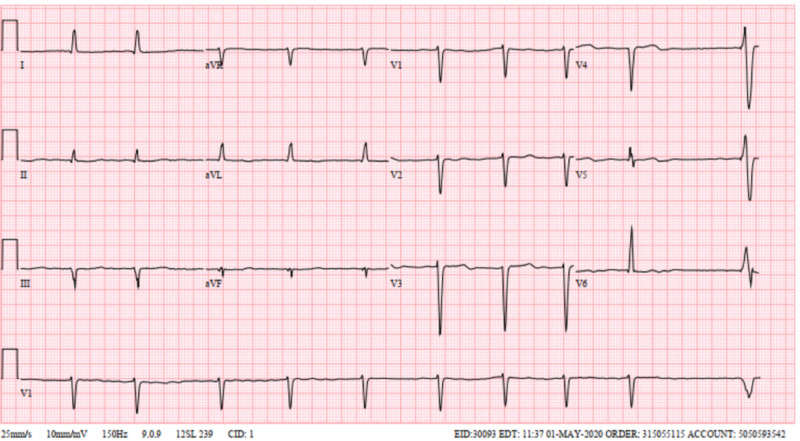
Electrocardiogram showing irregularly irregular rhythm consistent with atrial fibrillation

A 79-year-old male with a past history of nonischemic cardiomyopathy requiring implantable cardioverter-defibrillator (ICD), atrial fibrillation, hypertension, end-stage renal disease, and mild to moderate aortic stenosis now presented with lightheadedness and shortness of breath on mild exertion. The patient also had a history of atrial fibrillation for more than 15 years and developed intermittent episodes of GI bleeding requiring discontinuing and switching of different anticoagulation treatments. He was taking a reduced dose of apixaban with intermittent compliance before his current presentation. On admission, his vital signs were stable with a heart rate of 90 beats per minute and blood pressure of 110/83 mmHg. The cardiac exam revealed a late peaking systolic murmur at the aortic area with a soft second heart sound. No crackles, jugular venous distension, or pedal edema was noticed. Electrocardiogram revealed atrial fibrillation with slow ventricular response and occasional demand pacing (Figure 1).

**Figure 2:**
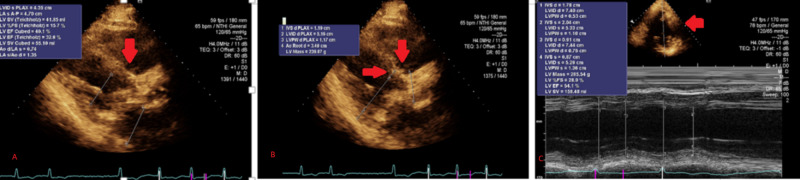
Echocardiogram showing severe aortic stenosis (A, B) Parasternal long-axis view with red arrows indicating a severely stenosed aortic valve with minimal opening in systole. (C) Echocardiogram with M-mode showing minimal opening of the aortic valve in systole and narrow valvular orifice consistent with severe aortic stenosis.

Cardiac troponin was normal. Hemoglobin was 9 g/dl (baseline anemic with a range of 8-10 g/dl). Echocardiogram revealed severe aortic stenosis with a valve area of 0.9 cm^2^ and mean and peak gradients of 37 and 72 mmHg, respectively, that were consistent with severe aortic stenosis (Figure 2).

**Figure 3:**
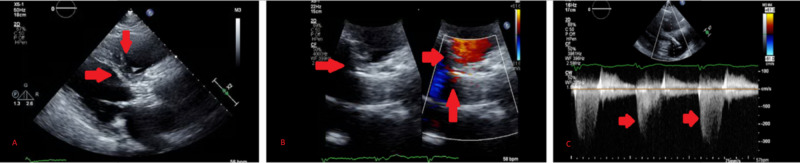
Echocardiogram post transcatheter aortic valve replacement (A, B) Parasternal long-axis view with red arrows indicating normally opening bioprosthetic aortic valve in systole which is also demonstrated by normal color flow Doppler. (C) Normal gradients across the replaced bioprosthetic aortic valve indicated by red arrows corresponding to a normally functioning valve.

On further history, he did endorse hospitalization for episodes of GI bleeding earlier the same month when a colonoscopy, esophagogastroduodenoscopy, and capsule enteroscopy were nondiagnostic. Due to persistent GI bleeding, his apixaban was discontinued despite having a CHADS2-VASc score of 5 but with a HAS-BLED score of 6 at that visit. The severe symptomatic aortic stenosis causing dizziness and lightheadedness combined with recurrent GI bleeding presumed to be related to the aortic stenosis leads to a decision of valve replacement procedure during the same hospital visit. Considering the patient’s age and high surgical risks (society of thoracic surgeons/STS score predicting a 13.3% risk of operative mortality), a transcatheter aortic valve replacement (TAVR) procedure was performed. Post-TAVR bioprosthetic valve replacement, gradients across the valve improved to mean and peak gradients of 6 and 14 mmHg, respectively (Figure 3).

This was followed by plans for placement of the Watchman device during the follow-up visit due to high bleeding risk and recurrent GI bleeds as well as high thrombotic risk and need for stroke prophylaxis.

## Discussion

Heyde’s syndrome is defined by the presence of severe aortic stenosis and GI bleeding with evidence of angiodysplasias and a reduced level of vWF factor in blood. In the absence of angiodysplasias on coloscopy or enteroscopy, a diagnosis of Heyde’s syndrome cannot be made. However, in patients with severe aortic stenosis, GI bleeding in the absence of angiodysplasias has been reported. These patients are referred to as ‘GI bleeding not related to angiodysplasias’ [6]. Our case was similar without identifiable angiodysplasias on endoscopy.

GI bleeding in Heyde’s syndrome results from an acquired form of vWF deficiency. vWF is essential to maintaining hemostasis and formation of platelet plug as well as plays a role in maintaining vascular integrity. It circulates in the form of multimers in blood and is cleaved by ADAMTS13 enzyme. When passing through the narrow aortic valve, the shear stress leads to abnormal unfolding of the vWF multimers. This exposes the A2 site where the ADAMTS13 usually binds resulting in cleavage of these multimers causing an acquired deficiency. Any form of sheer stress whether aortic stenosis or even hypertrophic obstructive cardiomyopathy can lead to this deficiency [7].

More than 60 years ago, this association was first described by Edward Heyde. Twenty-eight years after his discovery, angiodysplasias were identified as the source of GI bleeding in the majority of these patients [8,9]. Later studies not only confirmed the acquired deficiency of vWF multimers in these patients, but also revealed improvement in bleeding with increased levels of vWF multimers after valve replacement procedure. Surgical valve replacement is the gold standard treatment option for these patients. Data from observational studies also show that TAVR is a reasonable alternative in a subset of patients with high surgical risk [10,11]. Our patient underwent TAVR due to high surgical risks [12]. This was coupled with plans for placement of the Watchman device due to his high bleeding risk as predicted by his high HAS-BLED score and high thrombotic risk predicted by a high CHADS2-VASc score. One can argue that in patients with low risk of bleeding, the need for a Watchman device may not be necessary as the valve replacement usually leads to improvement in bleeding. However, the idea of TAVR coupled with Watchman device implantation in patients with atrial fibrillation to reduce their bleeding risks has been studied and the prospects seem to be encouraging [13]. 

## Conclusions

Patients with concomitant atrial fibrillation and aortic stenosis may present with recurrent GI bleeding due to anticoagulant use and presence of Heyde's syndrome, respectively. These patients may have symptomatic aortic stenosis and recurrent GI bleeds with high bleeding score on one hand. At the same time, their high thrombotic risk from atrial fibrillation usually necessitates some form of stroke prophylaxis. This can make the management challenging. A transcatheter valvular procedure coupled with plans for Watchman device placement may be performed for optimal management.
